# Use of a Probabilistic Motif Search to Identify Histidine Phosphotransfer Domain-Containing Proteins

**DOI:** 10.1371/journal.pone.0146577

**Published:** 2016-01-11

**Authors:** Defne Surujon, David I. Ratner

**Affiliations:** 1 Program in Biochemistry and Biophysics, Amherst College, Amherst, Massachusetts, United States of America; 2 Department of Biology, Amherst College, Amherst, Massachusetts, United States of America; Swiss Institute of Bioinformatics, SWITZERLAND

## Abstract

The wealth of newly obtained proteomic information affords researchers the possibility of searching for proteins of a given structure or function. Here we describe a general method for the detection of a protein domain of interest in any species for which a complete proteome exists. In particular, we apply this approach to identify histidine phosphotransfer (HPt) domain-containing proteins across a range of eukaryotic species. From the sequences of known HPt domains, we created an amino acid occurrence matrix which we then used to define a conserved, probabilistic motif. Examination of various organisms either known to contain (plant and fungal species) or believed to lack (mammals) HPt domains established criteria by which new HPt candidates were identified and ranked. Search results using a probabilistic motif matrix compare favorably with data to be found in several commonly used protein structure/function databases: our method identified all known HPt proteins in the *Arabidopsis thaliana* proteome, confirmed the absence of such motifs in mice and humans, and suggests new candidate HPts in several organisms. Moreover, probabilistic motif searching can be applied more generally, in a manner both readily customized and computationally compact, to other protein domains; this utility is demonstrated by our identification of histones in a range of eukaryotic organisms.

## Introduction

The explosion of genomic sequencing data in recent years enables in parallel the examination of an ever-growing number of encoded proteomes. As a result, investigators may find themselves exploring the predicted proteome of some newly sequenced organism of interest guided only by uncurated, machine annotation. While such automated annotation is of great use as a “first pass,” there is always the possibility of errors both of inclusion and omission, predicting incorrectly a specific function for a given protein, or failing to flag some protein as falling within a given functional grouping.

**Fig 1:**
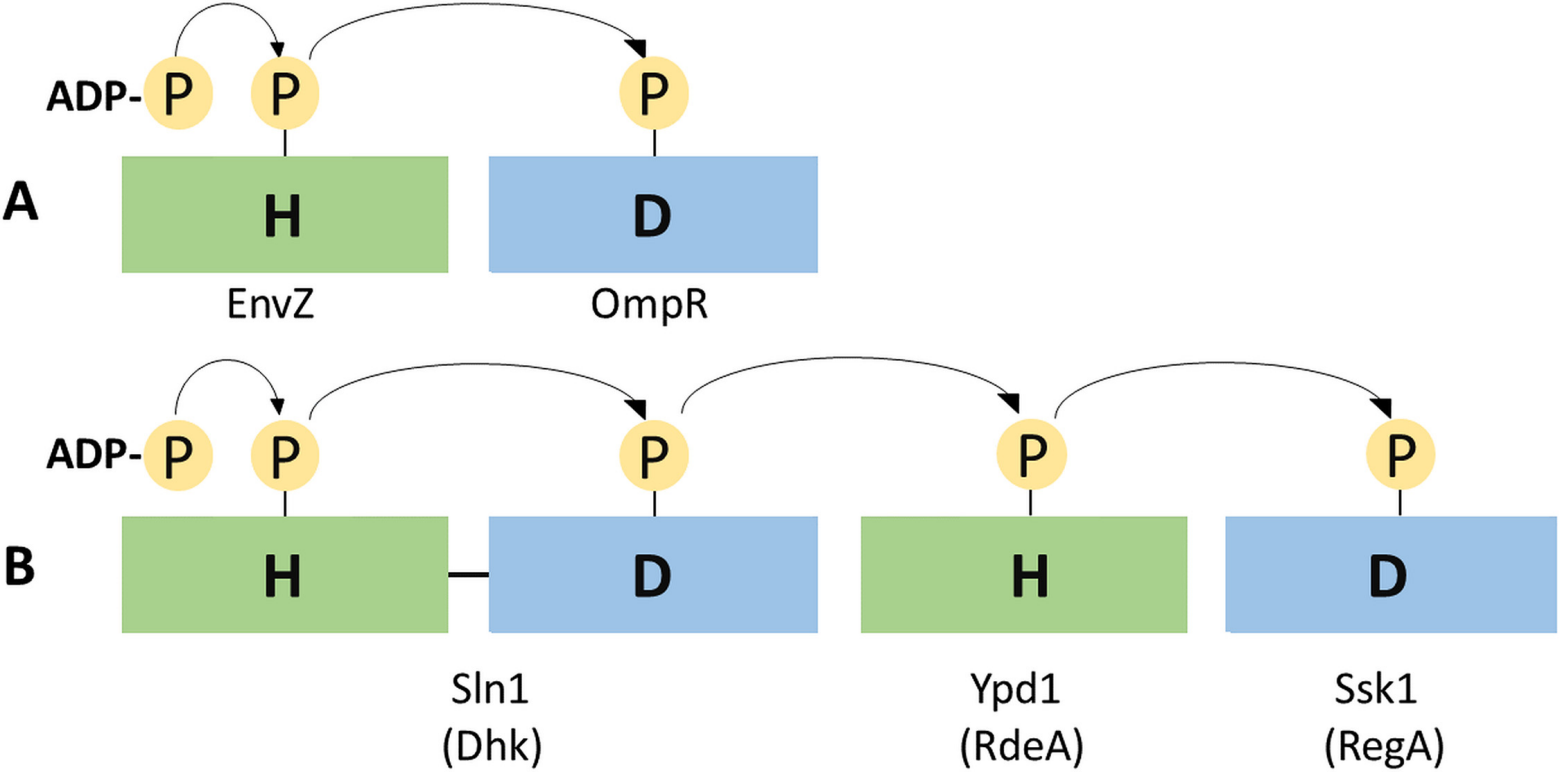
Architectural differences between His-Asp phosphotransfer systems. (A) Two component phosphotransfer involved in osmoregulation in *Escherichia coli* [1]. (B) Four component system in *Saccharomyces cerevisiae* involved in responding to osmotic stress, with the first two components on a single protein, Sln1. A similar architecture in *Dictyostelium discoideum* (in parentheses) responds to multiple histidine kinases and a range of initiating signals [2, 3].

Histidine phosphotransfer (HPt) proteins are components of the histidine kinase (HK) to response regulator (RR) signal transduction scheme. In bacteria, this type of phosphate transfer can typically be described as a “two component” signaling system, in which the phosphate moiety from a histidine residue on an autophosphorylating sensor kinase is relayed to an aspartic acid residue in the receiver domain of the RR protein, frequently a transcriptional activator [1]. In eukaryotes, and in some prokaryotic examples, a more elaborate transfer of phosphate occurs: while different molecular architectures are known, one common variant involves a “hybrid” histidine kinase (providing the first histidine, His1) that also includes a receiver domain (providing the first aspartate, Asp1) elsewhere in the same polypeptide. A distinct HPt protein provides the third phosphorylatable residue (His2), from which the phosphate is relayed to its final destination (Asp2) in the RR [2] (Fig 1). Greater complexity affords more opportunity for regulation, as well as a means to convey the signal between different cell compartments.

Importantly, phosphotransfer between histidine and aspartate residues is catalyzed by the receiver domains [1]. As HPt proteins are not known to have enzymatic activity themselves [4, 5], the evolutionary constraint on amino acid sequence divergence is not very strict, allowing HPt proteins to be small and the HPt signature motif itself, rather minimal. This makes it difficult to identify potential candidates of this protein family by traditional methods such as BLAST alignment. Ishige *et al*. [6] identified residues found in common among bacterial HPt domains, but the motif noted was of limited information content: Dxxxhxxxh**H**+h+GxAxxxGh, where the phosphorylated histidine is bolded; “D,” “G”, and “A” represent aspartic acid, glycine, and alanine, respectively; and where “x” represents any, “h,” hydrophobic, and “+,” positively charged amino acids. Recently, Fassler and West [5] noted an altered and expanded degree of homology among all four helices comprising fungal HPt proteins; the greatest conservation falls within the 18–20 residues spanning the helix containing the reactive histidine. Nevertheless, the identification of HPt (and other) domains remains challenging. We report here a relatively simple and generalizable search method for the identification of such domains based upon the likelihood of occurrence of amino acids in HPts flanking the key histidine residue. Plant and mammalian proteomes afforded datasets by which we tested our approach, since the former contain known HPts while the latter are presumed to lack such proteins. The simplicity of our method is a result of the incorporation of a Hidden Markov Model (HMM) with a linear topology, not tolerating insertions or deletions [7]. This, along with the Gibbs sampling algorithm [8] we use, makes our method particularly computationally efficient, with all of our results obtained on a personal computer in under five minutes. Among these results are novel sequences (in *Zea mays*, *Ajellomyces capsulatus*, *Mycobacterium tuberculosis*, and *Daphnia pulex)* that we have identified as containing the HPt motif. We then extend our approach, briefly, to the identification of histone proteins in diverse eukaryotes.

## Methods

### Determination of a Signature Motif

We define a motif by constructing a probability matrix P ∈ ℝ^20 x k^ from a reference sequence set, where k is the length of the motif as specified by the user. In the matrix P, the rows are indexed by the 20 naturally occurring amino acids and each column corresponds to the position within the motif to be constructed. Initially, we make a random selection of one substring of length k (k-mers) from each protein sequence present in the reference set, and store these k-mers in a separate array. For amino acid j and position 1≤i≤k, we define P[j][i] = (m_ji_+1)/M, where M is the total number of sequences in our reference set, and m_ji_ is the number of the selected k-mers having amino acid j at position i. We further define the likelihood of a given k-mer X = (x_1_,x_2_,…,x_k_), a sequence of k amino acids, occurring given the matrix P as

$$\varphi \left(X\right)=\text{Pr}\left(X|P\right)=\prod_{i=1}^{k}P\left[x_{i}\right]\left[i\right]$$ (1)

Since we use the amino acid frequencies in the reference set as their probabilities of occurrence in P when evaluating k-mers, we added a pseudo-count term of +1 to m_ji_ in order to avoid zero-likelihoods that could occur due to the relatively small size (on the order of 300 sequences) of the reference sequence set.

We then implemented the Gibbs algorithm to optimize the motif matrix P as follows: we select one of the M protein sequences in the training set at random. On this protein sequence, we make a pseudo-random selection of a new k-mer and update our stored k-mers by replacing the existing k-mer from this particular protein sequence with the new k-mer. Our pseudo-random selector weighs the likelihoods of every possible k-mer on the protein sequence by its calculated φ value. Thus, a k-mer from any protein that scores higher with the existing profile matrix P has a proportionally higher chance of getting selected. This way the Gibbs selection algorithm imposes a requirement that all k-mers forming the final profile matrix P have some level of agreement with each other and therefore reveal a conserved motif. Having updated our set of k-mers, we then update our P, and iterate this process N times. In each iteration, the Gibbs algorithm introduces some degree of randomness to ensure that a sufficient number of substrings are sampled from each protein. After a large number of iterations, the selected k-mers form a final matrix P which becomes less likely to change over subsequent iterations. We found it sufficient to set N = 2000 for a motif length of k = 20 and training sets containing ~300 protein sequences in the case of the Pfam HPt family [9], as the motif P had stopped changing before reaching the 2000th iteration, and the collected k-mers from each sequence were in as much sequence agreement with each other as possible. It’s important to select an appropriately large N when working with larger training sequence sets. As our reference set, we used the Pfam HPt domain family (PF01627), extracting either the “seed” subset or the subset of all eukaryotic entries. See “Results” below for discussion of our choice of these particular subsets.

The 2000-iteration process optimizes a profile matrix and yields a set of optimized k-mers, one from each sequence in the reference sequence file. In order to refine further the resulting motif and to avoid the output of a locally optimized P rather than a globally optimized P, this process was repeated 20 times and the highest-ranking profile of the 20 selected. To do this, the profiles were ranked based on how well each one of the k-mers in the final iteration agrees with the consensus sequence, which is defined by the k amino acids that are the most likely to occur at each position. The profile ranking algorithm selects the profile with the minimum number of mismatches between the consensus sequence and all selected k-mers.

Two other search algorithms are available as functions in the FIND_MOTIF.py file: Greedy (“main_greedy”) and Randomized (“main_randomized”) search. These search algorithms rely on finding the profile-most-probable k-mer of a given sequence rather than a pseudo-random sampling as is the case in the Gibbs algorithm. We define the profile-most-probable k-mer in a polypeptide sequence as the k-length subsequence k-mer that maximizes φ(k-mer). Randomized search is similar to Gibbs search, but after generating the initial profile randomly, it proceeds to select the profile-most-probable k-mer from each sequence again and reconstruct the profile matrix. Greedy search picks the first k-mer in the first sequence, constructs a profile based on this sequence, and iterates the process of picking the profile-most-probable k-mer from the following sequence (and updating the profile accordingly) for each sequence. This process is repeated for each k-mer in the first sequence. For the HPt motifs examined here, all three algorithms were equally effective in finding agreeing motif profile matrices in a span of minutes.

### Searching for a Motif in a Given Proteome

The motif search was done using the motif profile matrix P generated by the Gibbs Motif Generator algorithm as described above. Full proteomes were downloaded from UniProt [10]. Each protein sequence in the selected proteome was searched for the profile-most-probable k-mer given the profile matrix P. From this search, we recorded the k-mer with the maximal φ for each protein sequence in the proteome. This way, each protein is represented by the k-mer that scores highest (given profile matrix P) within the sequence of this protein. These k-mers were then ranked in a descending order according to their associated φ values. Outliers were defined in two different ways: the first definition (3IQR) identified all sequences within the queried proteome with φ exceeding Q3 + 3IQR on a logarithmic scale. The second definition (5% cutoff), more restrictive than the first in most cases, selected sequences within a proteome with φ scores greater than the 5th percentile φ obtained when the profile matrix P was applied to Pfam’s reference HPt dataset itself. This approach can be used when evaluating single protein sequences, or sequences that are not necessarily from the same proteome. Outliers, in either case, were then examined as possible HPt proteins. All python files and sample input files can be found at the repository: http://www.github.com/dsurujon/motifs/

## Results

**Fig 2:**
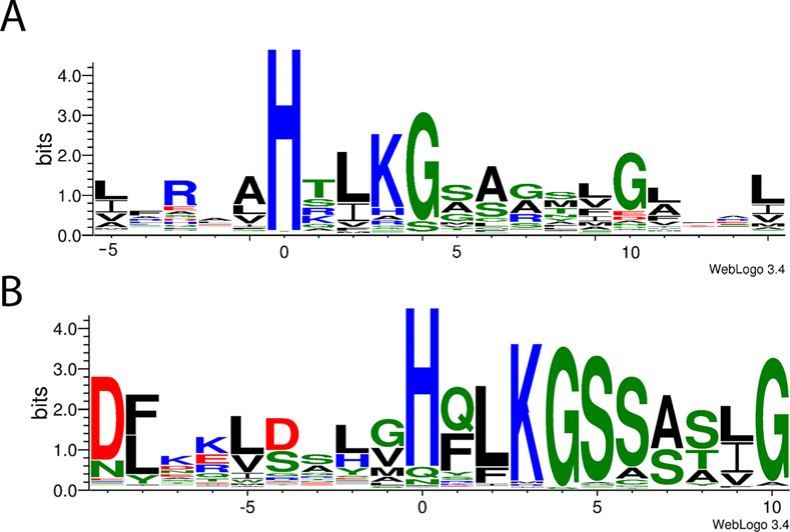
Representation of amino acid conservation flanking the reactive histidine of diverse HPt domains. In each logo, the height of individual columns reflects overall amino acid conservation (hence the information content) at that position, while the height of letters within a given column reflects the relative representation of the corresponding amino acid at that same position [14]. Amino acids are colored according to the scheme of [15]: hydrophobic residues are black; hydrophilic, green; acidic, red; and basic, blue. In (A), the motif was derived from the 241 seed sequences, mostly prokaryotic, used to generate PF01627; in (B), all 344 eukaryotic members of the complete family were analyzed to generate the motif. Note that the search algorithms employed identify regions with the highest overall conservation within the two different sequence sets, and therefore output motifs that situate the phosphorylated histidine (residue “0”) at different relative positions. Furthermore, the phosphorylated histidine is not absolutely conserved in the eukaryotic sequences presented. This is due to certain proteins (such as AHP6; see text) that lack a histidine at the conventional position but are included in PF01627 nevertheless; their biological function may be related to but distinct from phosphotransfer per se.

Although a four helix bundle is the well-conserved structural feature of known HPt proteins [11, 12, 13], the evolutionary constraints upon amino acid sequence within that bundle appear minimal: with the exception of the phosphorylated histidine, no nearby position is absolutely conserved among either bacterial or eukaryotic entries amongst the seed sequences used to generate the Pfam HPt family. Thus it is challenging to identify possible HPt proteins in an uncharacterized proteome. As described in Methods, we employed Gibbs sampling along with a motif searching algorithm to identify regions of twenty contiguous residues encompassing the key histidine and other residues likely to be near the RR-interaction surface. The region chosen spans the best conserved portion of the longer (85 residue) Pfam motif and is computationally more tractable than a larger profile matrix would be. Initially, the members within Pfam’s “seed sequence” subset formed the basis of our search. The great preponderance of the HPt family members (99.1% of 42,599 sequences as of June 2015) are prokaryotic, and the seed subset is similarly biased; the resulting logo presented in Fig 2(A) thus reflects mostly prokaryotic conservation. Because of our ongoing interest in signal transduction within the amoeba *Dictyostelium discoideum* [3], we generated as well a motif derived solely from the smaller number of eukaryotic sequences in PF01627, as shown in Fig 2(B). Other than the histidine residue itself, it is apparent from either logo that sequence conservation within this helical stretch of the HPt domain–bacterial or eukaryotic–is limited, as others have long appreciated [1, 5, 6].

Despite the lack of absolute residue conservation in the vicinity of the reactive histidine, the observed likelihoods of occurrence of amino acids in the region can still enable an assessment as to whether or not an uncharacterized protein is likely to function as an HPt protein. The motif profile matrix assigns a likelihood of occurrence (φ) to each k-mer within a target polypeptide, i.e. it calculates the probability of the generic HPt motif being exemplified by the particular amino acid sequence found in every k-mer. (Note that, in contrast to an expectation or E value, the likelihood defined here is greater for k-mers more similar to known HPts). By identifying the highest scoring k-mer for each polypeptide in a given proteome, and ranking all proteins within that proteome according to the likelihood scores (φ) of these selected k-mers, it should be possible to identify presumptive HPt proteins from any organism for which a proteome is available.

**Fig 3:**
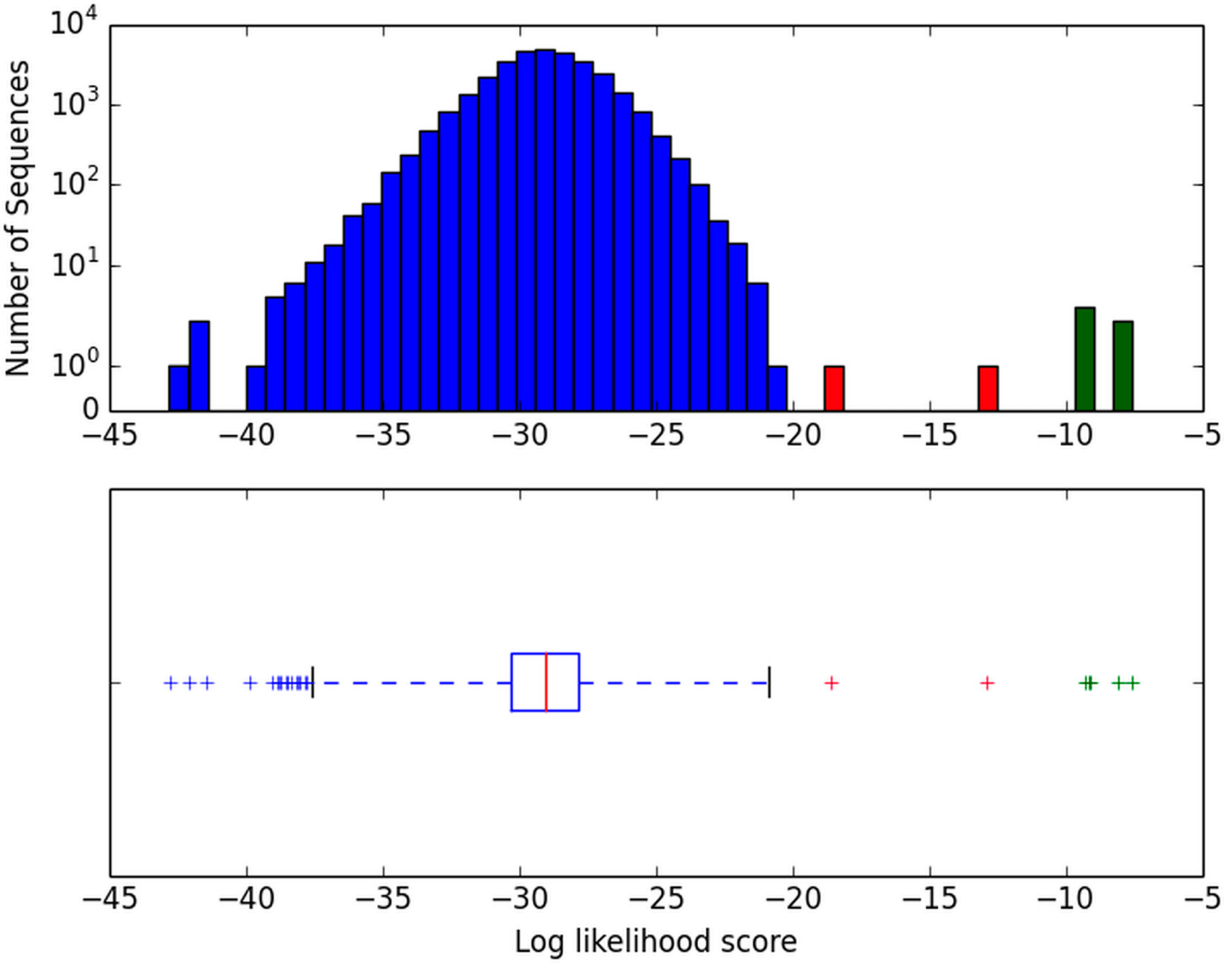
φ score distribution in the *A. thaliana* proteome. Upper: Histogram representation of the logged distribution of proteins in the *A*. *thaliana* proteome as a function of their likelihood of occurrence in the eukaryotic HPt motif profile matrix. Note the several proteins with likelihood scores orders of magnitude greater than those of the bulk distribution. Lower: The same log likelihood scores of the *A*. *thaliana* proteome replotted as a box plot. The Q1-Q3 interquartile range (IQR) is boxed with the median (Q2) shown in red; whiskers indicate 3IQR limits. The five most prominent high-scoring outliers, as well as two other proteins significantly closer to the bulk distribution, are again indicated (“+”) in green and red, respectively. Additional information about each of the seven outlying proteins (including duplicates of three entries) appears in Table 1 and Supporting Information S1 Table.

Plants typically contain a multiplicity of histidine phosphotransferases. Among the plants, *Arabidopsis thaliana* is perhaps the most thoroughly investigated species–both *in silico* and experimentally. Thus we tested the validity of our approach by searching the well-characterized *A*. *thaliana* proteome with our motif matrix. Fig 3 (top) shows the ranked results of such analysis of all 31,548 proteins in the proteome. The distribution of likelihood scores is very wide, ranging over 30 orders of magnitude. Given the length of the HPt motif and the laxity of sequence conservation across it, the absolute likelihood assigned to any candidate HPt domain must be small (generally 10^−8^ or even far below). The median likelihood of a randomly selected *A*. *thaliana* protein’s profile-most-probable k-mer being generated by the eukaryotic HPt motif profile matrix is expectedly miniscule (φ = 3.1*10^−29^), as is evident from the flanking box plot interquartile range (IQR) shown in the lower panel. (There are, after all, in excess of 10^26^ potential 20-long sequences that can be generated by the set of 20 standard amino acids.) The right hand 3IQR box plot “whisker” in Fig 3 extends to a likelihood of 10^−21^. Notably, beyond that whisker, seven proteins contain k-mers much more similar to the eukaryotic HPt motif (of Fig 2B) than do all other *A*. *thaliana* proteins. These seven “outliers” at the upper end of the logged likelihood distribution, each listed in Table 1, merit individual consideration.

**Table 1:** Examination of presumptive HPt domain-containing proteins in *A*. *thaliana*.

Rank	φ	Uniprot ID	Protein Name	Motif found as	Motif	Pfam	SMART	InterPro	PROSITE	Reference
1	2.73E-08	Q9ZNV9	AHP1	DFKKVDPHV**H**QLKGSSSSIG	+	+	+	+	+	[16]
2	8.33E-09	Q8L9T7	AHP5	DFKQVDSGV**H**QLKGSSSSVG	+	+	+	+	+	[16]
3	8.69E-10	F4J1I8	AHP4	DFNRLDSYM**H**QFKGSSTSIG	+	+		+	+	[17]
5	7.09E-10	Q9SAZ5	AHP3	DFKLVGSSV**H**QLKGSSSSVG	+	+	+	+	+	[18]
6	5.15E-10	Q9ZNV8	AHP2	DFSQVGASV**H**QLKGSSSSVG	+	+	+	+	+	[16]
7	1.33E-13	Q9SSC9	AHP6	DYKKIGLHLNQLVGSSSSIG	+	+		+	+	[19]
9	2.68E-19	F4K2D8	LETM1-like	ALVRLESLLQQLHASSSSSG	+					
11	1.33E-21	F4K740	Uncharacter-ized protein	DYQKLSETGNAVKGSSSNKR						
12	1.05E-21	O49508	F-box protein At4g18380	VFKPLQALGQFLKRSGSSSL						
13	9.90E-22	Q93YS4	ABCG22	ELEEVSSGAALSRASSASLG						

The ten highest scoring unique proteins in that proteome (UP000006548) are considered in detail, while duplicates, possibly due to splice variants, of three proteins (AHP4, AHP6, LETM1-like) with identical motif sequences and scores were removed. (For additional information, see Supporting Information S1 Table.) The higher the likelihood, the greater the similarity of the sequence shown to the canonical, weighted motif. In the sequences shown, the reactive histidine is designated by **H** and other histidine residues, unlikely to serve that role, by H. (Histidine residues displaced from the usual position within the α-helix by two or three residues are expected to be oriented incorrectly and as a result fail to interact with catalytic residues in a postulated receiver domain partner. This was demonstrated *in vivo* by Chang *et al*. for *D*. *discoideum’s* RdeA protein [20]; see also S1 Table.) Of the ten proteins shown, the known phosphorelay proteins AHP1-5 are orders of magnitude more similar to the canonical eukaryotic HPt motif than all other *A*. *thaliana* proteins. See Results for a discussion of AHP6 and the LETM1-like protein (both of which contain only out-of-position H residues). The final three entries (ranks 11–13) fall within the 3IQR whisker, and have correspondingly little resemblance to *bona fide* HPts. They are included here merely to suggest the increasing divergence from the HPt motif of all the other thousands of *A*. *thaliana* proteins. + indicates that a protein exceeds our 3IQR motif threshold or, for comparison, is identified as an HPt in the other four databases shown. Except for the absence of AHP4 and AHP6 from SMART and the spurious highlighting of LetM1-like by its motif matrix resemblance, agreement for the various methods is complete. References are to functional characterizations of the previously designated AHP proteins.

The five *A*. *thaliana* proteins with greatest similarity to the eukaryotic motif, as ranked in Table 1 and denoted in green in Fig 3, are precisely the five proteins previously determined by others to be functional Arabidopsis histidine phosphotransferases, as reflected in their established “AHP” designation [21]. They are clear outliers from the 3IQR whisker cutoff we employ, with φ values ranging from 3x10^-8^ to 5x10^-10^. The next highest scoring outlier (shown in red in Fig 3 and ranked 7^th^ in Table 1) affords an interesting case: while AHP6’s name might suggest it is also an Arabidopsis HPtase, its divergence from the canonical motif (φ = 1x10^-13^) is considerably greater than those of the five higher scorers. In fact, AHP6 is not a functional HPt, acting instead as an inhibitor of *bona fide* HPt signal transduction [19, 22]. Its sequence presumably reflects an origin via duplication of and evolutionary divergence from some ancestral HPt gene, consistent with the reduced motif occurrence likelihood we report. Note further that, while AHP6 contains a single histidine residue (H) within its best scoring k-mer, that histidine is not in the optimal position within the helix to serve as a phosphorylation substrate/donor. Rather than protruding from the conserved four helix bundle so as to interact with receiver domains of HK and RR partners [12], the histidine residue of AHP6 would be expected to face inwards, towards the other helices of the bundle. Site-directed mutagenesis of an HPt protein in another species confirms the point: the RdeA protein of *D*. *discoideum* contains a histidine residue at the canonical position within the helix as well as another histidine two residues earlier in the sequence. Chang *et al*. established that the conserved histidine is essential for RdeA function, while mutation of the other histidine had no discernible effect upon *in vivo* phenotype [20]. See Table 2 and especially S1 Table for the RdeA motif sequence.

The only other *A*. *thaliana* outlier identified, scoring even closer (φ = 3x10^-19^) to the 3IQR cutoff, is the hypothetical protein “LETM1-like.” It also lacks a histidine at the appropriate position, and we do not expect it to function as an HPt. For illustrative purpose, Table 1 also includes the next three highest ranked Arabidopsis proteins, these all falling within the 3IQR whisker, all lacking a histidine residue in their profile-most-probable k-mers, and none of them expected to be phosphorelay proteins. Thus the probabilistic motif search method we describe has correctly identified the five true HPts in *A*. *thaliana*, quantitatively distinguishing them from the related HPt inhibitor AHP6, and even more so from all other entries in the complete proteome.

**Fig 4:**
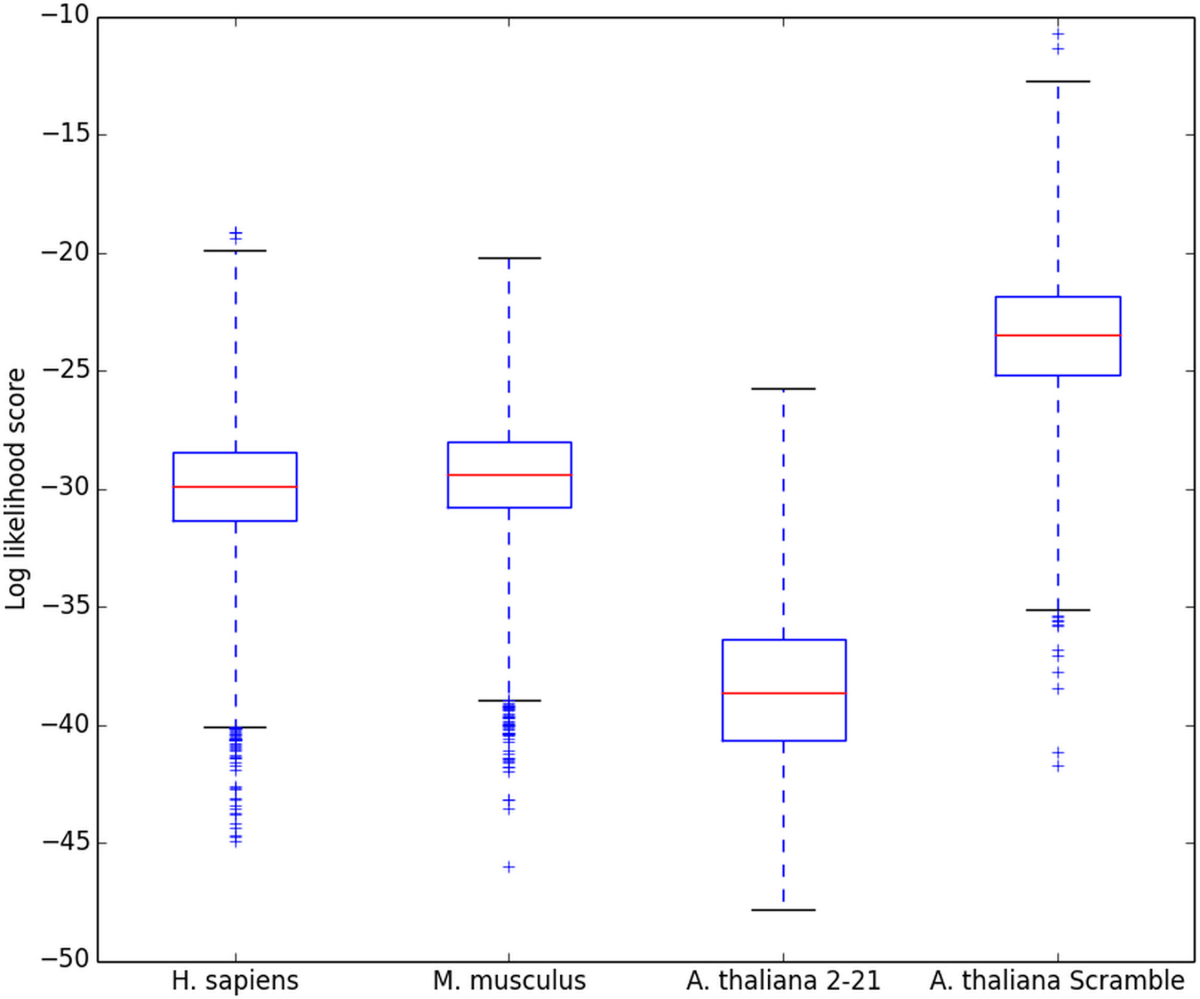
Testing of false positive identifications. The HPt motif likelihood distribution was examined for the human and mouse proteomes, a pseudo-random selection of 20 amino acid-long sequences (residues 2–21 for all proteins in the *A*. *thaliana* proteome), and a search of the *A*. *thaliana* proteome with a column-scrambled motif. Ideally, no HPt domains would be indicated in any of these searches. The Q1-Q3 IQR and whiskers are indicated as in the previous figure. The few false positives detected, evident in the first and fourth box plots, exhibit very low likelihood scores barely above the 3IQR cutoff.

Metazoans are not known to encode any HPt domains [23]. While limited phosphorylation of histidine residues has been reported in mammals [24], the mechanism of modification seems distinct from that in the HK to RR pathway. To examine the incidence of false positive hits generated by our search method, we examined the proteomes of two well-studied mammalian species, humans and mice, for similarities to our eukaryotic query motif. Fig 4 reveals that, while there are two distinct proteins in humans with φ values just above the 3IQR whisker threshold, their likelihood scores are very low (φ≈10^−20^), and there is little reason to suspect them of functioning as HPts. Indeed, each lacks any histidine residue in its profile-most-probable k-mer. (See S1 Table.) No outliers are seen in the mouse proteome, as expected. Additional negative controls were performed by two modified searches of the *A*. *thaliana* proteome. Rather than first searching each protein for its highest scoring 20-long k-mer as we usually do, we instead confined the analysis to the 20 amino acids (“2–21”) immediately following an initiating methionine: as expected, no HPt is found within this artificially restricted subset of the plant’s proteome. Finally, we shuffled the columns of the profile probability matrix while maintaining the relative representation of amino acids at individual positions within our eukaryotic HPt motif. Searching the complete *A*. *thaliana* proteome with one hundred such scramblings and matrix searches resulted in an overall false positive rate of 0.0087% across the *A*. *thaliana* proteome, consistent with the example shown in Fig 4 with its two low-scoring outliers. Clearly these outliers need have no relation to any real HPt. Collectively, these four controls reveal that our method retrieves only a small number of false positives in datasets lacking *bona fide* HPts. We think it more helpful to capture a few additional proteins that can then be considered, and perhaps excluded, individually rather than to raise the discrimination of our 3IQR cutoff, in which case legitimate HPt domains might be overlooked.

**Fig 5:**
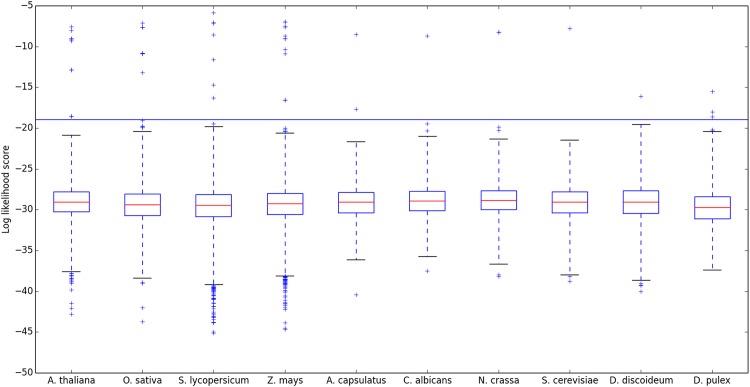
Logged motif likelihood score distributions of select eukaryotic proteomes. Four plant species are followed by four fungi, a protist, and an arthropod. Full proteomes were downloaded from UniProt and their motif likelihood distributions calculated. IQRs and whiskers are as before. The blue horizontal line corresponds to an alternative approach to identifying outliers (“5% cutoff” as explained in Methods) which is independent of the proteome examined. Outliers are enumerated in Table 2, with full details of each candidate given in S1 Table.

Having established the ability of probabilistic motif searching to detect HPt proteins while generating only a modicum of false candidates, we turned our attention to a small sampling of diverse eukaryotic species, including several plants and fungi. Higher plants typically encode many HPt proteins, some of which are involved in pathways modulating cytokinin signaling [25], light signaling [26], and possibly ethylene signaling [27]. Fungi and protists, in contrast, typically contain far fewer HPt proteins, and often only one [5]. Fig 5 reveals that, simply by motif score, numerous HPt domains are predicted not only in *A*. *thaliana* but also within the encoded proteomes of the three other plants shown, *Oryza sativa*, *Solanum lycopersicum* and *Zea mays*. From the figure, its enumeration in Table 2, and the details of all outliers as provided in S1 Table, several generalizations can be made. To begin, the outliers span a range of likelihood values, with the high scorers surely representing legitimate HPt proteins or domains, but those much closer to the 3IQR whisker often appearing anomalous in ways likely to preclude their functioning in phosphorelay. In the *O*. *sativa*, *S*. *lycopersicum*, and *Z*. *mays* proteomes, candidates with log φ = -20 are clearly bogus, lacking the reactive histidine and/or other highly conserved residues. To reduce the number of false positives to be examined, we also employed in this instance a more restrictive cutoff (“5%”) better reflecting the best conserved components of the Pfam HPt training set (see Methods). That criterion eliminates some but still not all false positive candidates, reflecting the unavoidable need for investigators to examine individual hits with some care. (For *A*. *thaliana*, the two cutoffs do not differ in the outliers identified, but for other species here, the 5% criterion is, in several cases, the more convenient.) As with *A*. *thaliana* previously, the probabilistic motif search is able to identify all of the HPt proteins included in any of the other databases, Pfam, SMART, InterPro, and PROSITE, for all three additional species. Moreover, there are also examples where the motif method highlights proteins overlooked in existing databases. An intriguing case is that of the *Z*. *mays* hypothetical protein A0A096TIV3, with φ = 1x10^-11^ (details in S1 Table). Its high likelihood score reflects excellent conservation of the best conserved residues of the motif in Fig 2(B). BLAST analysis of the full length 232 residue polypeptide reveals homology to other maize HPt proteins, especially to ZmHP2 [12]; while homology is best across the 20-long string we initially identified (as expected, given the laxity of HPt conservation elsewhere), in fact alignment extends for an additional 40+ residues on one or other side. Lesser homology can be found with maize HPt proteins 1 and 5. Of course confirmation or exclusion of this candidate as a newly discovered, *bona fide* maize HPt will require considerable additional work, including biochemical investigation.

Fungi present a much simpler situation, with far fewer HPt candidates evident in Fig 5 and Table 2. For *Saccharomyces cerevisiae*, motif searching yields the same, single HPt (YPD1) long known [5]. The method gives a correspondingly clean and simple result for the YPD1 homologs found in *Candida albicans* and *Neurospora crassa*, once the two relatively low scoring hits in each proteome (with likelihoods all falling between our 3IQR and 5% cutoffs) are quickly excluded by simple examination of their k-mer sequences. (See also S1 Table.) Motif likelihood scoring reveals two outliers in the *Ajellomyces capsulatus* proteome: the first, with φ = 3x10^-9^, is a clear YPD1 homolog that also appears in the four databases; the second hit is more intriguing, as it is absent from existing databases but scores above both cutoff criteria (φ = 2x10^-18^). This protein, F0UAC2, was previously annotated as being a member of a four component signaling pathway but predicted to be a hybrid histidine kinase comprising catalytic, dimerization/phosphoacceptor, and receiver domains. Based on its φ score and k-mer sequence, we propose that this large polypeptide may also contain a distinct N-terminal HPt domain. Such tripartite architecture is reminiscent, among other systems, of the BvgS protein of *Bordetella pertussis* [2] but has not been identified previously in fungi [5].

**Table 2:** Comparison of the number of HPt domains revealed by probabilistic motif searching with their representation in several widely used protein databases.

Species	Proteome ID	Motif (3IQR)	Motif (5%)	Pfam	SMART	InterPro	PROSITE
Arabidopsis thaliana	UP000006548	7	7	6	4	8	6
Oryza sativa	UP000000763	9	5	5	1	5	2
Solanum lycopersicum	UP000004994	8	7	6	3	7	6
Zea mays	UP000007305	10	7	5	3	6	4
Ajellomyces capsulatus (Strain H88)	UP000008142	2	2	1	1	1	1
Candida albicans	UP000001429	3	1	1	1	1	1
Neurospora crassa	UP000001805	3	1	1	1	1	1
Saccharomyces cerevisiae	UP000002311	1	1	1	1	1	1
Dictyostelium discoideum	UP000002195	1	1	1	1	1	1
Daphnia pulex	UP000000305	5	3	1	1	1	1
Homo sapiens	UP000005640	2	0	0	0	0	0
Mus musculus	UP000000589	0	0	0	0	0	0

Data in the Motif (3IQR) column reflect the number of outliers in each proteome above the 3IQR threshold set as described previously. The Motif (5% cutoff) column shows the number of proteins with φ scores above an alternative, constant threshold, 1.07E-19, determined as described in Methods. Duplicate entries (sequences with identical scores, motif k-mers, and mapped genes) were removed from the tabulation; they may represent splicing variants or, alternatively, redundant listings. Details for each entry tabulated (including duplicates) are to be found in Supporting Information S1 Table. F0UAC2, the additional HPt domain detected in the *A*. *capsulatus* proteome by motif searching, is a histidine kinase. The sequence A0A096TIV3 in the *Z*. *mays* proteome has no annotations in any of the four databases. Its high φ score (1.35x10^-11^) suggests that this sequence may represent a novel HPt protein in this plant. The top HPt candidate (E9I376) detected by our motif search of the *D*. *pulex* proteome is intriguing: though it is an outlier with a φ score more than two orders of magnitude higher than the second highest score, no HKs or RR proteins are known in this species or, for that matter, known to function in other animals. There is, unfortunately, no experimental evidence concerning E9I376 function, even though it is identified as an HPt protein by all approaches above.

We examined a small number of proteomes from other eukaryotic phyla. In the proteome of the amoeba *Dictyostelium discoideum*, the organism that originally attracted our attention, only a single HPt domain protein can be detected. That protein (shown in Fig 5 with φ = 8x10^-17^; see also Table 2 and S1 Table) is the well-studied histidine phosphotransferase RdeA [20, 2]; there is no other protein with a motif similarity score within five orders of magnitude. Similarly unique HPt proteins, homologous to RdeA, can be found within the proteomes of related Dictyostelids including *D*. *fasciculatum*, *D*. *purpureum*, and *Polysphondylium pallidum* (data not shown). Insects are not known to contain any HPt proteins or domains. Motif searching of several insect proteomes (including *Drosophila melanogaster*, *Aedes aegypti*, and *Camponotus floridanus*) returned the expected negative result, but we were surprised to see several probabilistic outliers in the proteome of a crustacean, namely the water flea *Daphnia pulex*. The striking outlier with φ = 3x10^-16^ (Fig 5), encoding a small (137 aa) polypeptide of size not unlike that of other free-standing HPtases, is denoted in all the databases in Table 2 as an HPt protein. In addition, two more candidates score above both motif probability cutoffs we have employed (Table 2), though with much reduced likelihood values; see S1 Table for details. HPt domains are not known in metazoans. Whether any of these hits of hypothetical proteins are real, perhaps reflecting horizontal gene transfer, or instead derive from errors in construction of the *D*. *pulex* proteome or are merely fortuitously homologous to our motif, is unknown at present.

Table 2 compares, for each species included, the results of our probabilistic motif search with the number of HPt domains to be found in several widely used protein databases, namely SMART [28], Pfam [9], InterPro [29], and PROSITE [30]. Employing the 5% cutoff for the designation of motif probability outliers, there is perfect agreement for half the species shown: *C*. *albicans*, *N*. *crassa*, *S*. *cerevisiae*, *D*. *discoideum*, *H*. *sapiens*, and *M*. *musculus*. However, there are many instances in which one database or another is incomplete; conversely, in some cases a database will include duplicate entries. The SMART database clearly overlooks validated or at least presumptive HPt domains for each of the plants shown. Pfam, providing us the reference sequence set, is in closer agreement with our results, and unsurprisingly our motif searcher does not omit any sequences identified by Pfam. In fact, we present a number of sequences apparently omitted in the Pfam HPt family. With the exception of a *Z*. *mays* sequence, PROSITE generally performs similarly to Pfam. However, there are evident omissions in the *Oryza sativa* proteome in the PROSITE database. This might represent organism-specific artifacts in the assembly of the database rather than an overall defect in identifying HPt proteins, as *O*. *sativa* is the only proteome above with multiple omissions. InterPro, by pooling data from multiple sources as well as running its own algorithm [31], is the most complete database of the four but contains duplicates in the form of fragmented sequences (as in the case of *Z*. *mays*). Moreover, some entries in InterPro are likely erroneous, for example the two *A*. *thaliana* entries included in InterPro but none of the other databases, and with no high scoring k-mers whatsoever (S1 Table). Our motif method generally agrees well with results tabulated in InterPro but may in fact be the more informative in some cases: the motif method identifies an extra HPt candidate in the proteomes of *A*. *capsulatus and Z*. *mays*, and calls attention to the possibility of such protein(s) existing even in the crustacean, *D*. *pulex*. See the Table 2 legend for more discussion of various disparities and Supporting Information S1 Table for a detailed listing of individual HPt candidates identified by one or more approaches.

To test the generality of our method of motif discovery, we turned to a completely different, widespread protein class. The Supporting Information (S1, S2 and S3 Figs and S2 Table) demonstrates that a probabilistic motif search can also be used to identify histones from diverse eukaryotic proteomes. A motif was generated with the core histone family (Pfam ID: PF00125) as training set, comprising histone types H2A, H2B, H3 and H4, but excluding linker histone type H1. The 15-mer motif logo (S1 Fig) is common to all four core histones, and appears to be located in functionally relevant areas of the nucleosome, mainly in regions of histone-histone interaction and histone-DNA binding (S2 Fig) as revealed by VMD software [32]. A more detailed examination of the highest ranking protein sequences (S3 Fig) from the well-annotated proteomes of six organisms reveals that proteins identified by our motif finder (underlined entries in S2 Table) are in general agreement with those that have been previously characterized and/or annotated as histones. This demonstrates the generalizability and flexibility of our motif-based search algorithm.

## Discussion

The recognition of functional motifs within protein datasets can pose a serious bioinformatic challenge, especially when there are only one or a few amino acid residues essential for function, with flexibility at other positions. In the case of histidine phosphotransferase (HPt) domains considered here, there is the requirement for a histidine positioned centrally located within an alpha helix (of a four helix bundle), but limited evolutionary sequence conservation beyond that. To approach this challenge of identification, we first created a probability matrix reflecting the proportion of each of the 20 amino acids at every position in the vicinity of the reactive histidine. Then, for every protein within a proteome of interest, we searched for its profile-most-probable k-mer, i.e. the contiguous stretch of amino acids in that protein most likely to exemplify the weighted, canonical HPt motif. Finally, we ranked all members of the proteome based on their profile-most-probable k-mers in order to look for proteins with the most probable HPt signature. As shown in Figs 3 and 5 and in Tables 1 and 2, examination of the range of motif matrix similarity scores does reveal known, functional HPt domains in several plants, fungi, and protozoa, quantitatively distinguishing these HPts from other proteins within a given proteome. This also demonstrates that 20 amino acids constitute a sufficient length for the HPt motif. When applying this method to other protein families, an appropriate motif length should be selected based on structural and functional inferences. For example, a motif of length 15 is enough to identify structurally relevant portions of all members of the histone family in animals and yeast (see S2 Table).

It is of course inappropriate to accept a designation of function merely from a motif similarity score; the negative controls presented in Fig 4 reveal a small number of false positives inappropriately highlighted by our search criteria. Nevertheless, researchers can examine outlying proteins individually in order to appreciate the sequence responsible for their high scores before exploring other, experimental avenues to assess function. Conversely, one must appreciate that a sufficiently novel HPt, a “pioneer” dramatically different in sequence (and presumably also structure) from others known to date, may well be overlooked by our method, as it would by others that are homology-based.

We have compared the results of the search method described here with those obtained by querying four commonly used databases, SMART, Pfam, InterPro, and PROSITE. In general, our results agree closely with the tabulations in InterPro. In contrast, the SMART listings appear sometimes to be incomplete, presumably again reflecting the use of different assembled proteomes and highlighting the importance of reference proteome databases such as that assembled by UniProt. Our motif search avoids the duplications and omissions in each of the four comparison databases as it is employed directly on a single (presumably most recent version of the organism’s) proteome. At the same time, our motif probability matrix has detected a new, candidate HPt protein or protein domain in the *Z*. *may*s and *A*. *capsulatus* proteomes, and, unexpectedly, at least one such protein in *D*. *pulex*. Of course whether such discovery might have been anticipated, as in maize, or is surprising (in the case of a crustacean), no conclusion as to biological function can be drawn without actual experimentation.

While the φ scores presented here concern eukaryotic HPt domains, a similar searching of prokaryotic proteomes is similarly possible using the motif matrix profile undergirding the logo in Fig 2(A). The results of such investigation of several bacterial species (not shown) are in general accord: again, our motif search identifies HPt domains in good agreement with those tabulated by InterPro. Worthy of note is the hypothetical protein L7N4Q0 in *Mycobacterium tuberculosis*. Although it is predicted to be an Acyl-CoA dehydrogenase by Pfam, InterPro and PROSITE, this sequence is a clear outlier by our analysis: it has a motif φ of 2.91x10^-17^ vs. the 3IQR whisker cutoff within the proteome of this organism at 5.32x10^-19^. This suggests a second possible function, histidine phosphotransfer, of this protein which might ultimately be verified by biochemical assay. While *M*. *tuberculosis* is known to employ two-component histidine kinase signaling [33], L7N4Q0 would be, if confirmed, the first example of an HPt protein in this species, suggesting the possibility of even more complex histidine kinase-dependent regulation.

The approach we have employed is not limited to HPts but can be applied to other domains exhibiting even modest conservation. We demonstrate this generalizability by identification, as presented in the Supporting Information, of a motif (S1 Fig) shared by the four types of core histones (S3 Fig and S2 Table). This motif also appears to have functional relevance, appearing in either histone-histone or histone-DNA interaction surfaces in the different histone types (S2 Fig). In fact, more sophisticated and computationally demanding implementations of the same principles of motif identification have been used for the discovery of transcription factor binding sites [34] and promoter elements [35].

The detection of motifs, in nucleic acids or proteins, often employs hidden Markov models. The motif finding algorithm employed by Pfam is HMMER [36]. This method (also available as a software package) resembles our profile-dependent HMM-finding algorithm; in order to simplify the HMMER algorithm and make it more time-efficient, Eddy has excluded indels in the HMM map, as is also the case in our algorithm. The earlier implementations of HMMER used the relatively costly Viterbi algorithm and are prohibitively slow in some applications. The 2011 update by Eddy, HMMER3, employs a bayesian Forward-Backward algorithm to replace the Viterbi method for the sake of efficiency. The costliness of the Viterbi algorithm is due to the fact that it is an exhaustive method, evaluating each possible transition from any possible state (each of the 20 amino acids) in the preceding position to any possible state in the current amino acid. In our model, we omit the transition probabilities in order to simplify our approach (employing the Gibbs sampling method). This omission and the short length of our motif result in the memory requirements for our motif finder being minimal. On a representative run of the algorithm for the identification of HPt motifs, the python script used less than 30MB of memory.

The motif consensus utilized by PROSITE, on the other hand, relies on a pattern of either specific residues or the possibility of multiple residues (at a given position) but without weighted likelihoods of occurrence. This and other such rigid approaches offer only limited information, a subset of that offered by methods incorporating an HMM. While it may be sufficient to employ the PROSITE sequence search with multiplicity in some cases, for protein families such as the HPt family, the HMM-based motifs are more informative. The PROSITE database does provide profile matrices for protein families, but the construction of these matrices relies on generating a multiple sequence alignment, and the profile matrix spans the entire protein. The generalized profile used by PROSITE differs from HMM-profile matrices in that generalized profiles are not based on formal probability theory. In our approach, we avoid the need for a pre-aligned set of sequences, and show that a much shorter motif is sufficient for the identification of HPt and histone proteins.

Our algorithm can also be customized to introduce certain constraints relevant to the protein family being examined. For example, rather than starting the Gibbs sampling with a completely random collection of k-mer sequences, a biased motif profile P’ with an absolutely conserved histidine residue at a specified position can be taken as an input and used for the following rounds of pseudo-random sampling. The HMM underlying our approach is relatively simple, as it accounted for all possible amino acid substitutions within a contiguous stretch but allowed no amino acid insertions or deletions. In the case of the HPt domain, excluding the possibility of indels ensured the preservation of the relative position of important residues within the histidine-containing helix of the four helix bundle. For other domains, whether indels should be allowed or can safely be excluded will reflect the particular molecular interactions involved. When indels are excluded, sequences do not have to be pre-aligned for their analysis. Moreover, the absence of indels, reducing the number of nodes 3-fold and the number of transitions 9-fold in the Markov chain, allows for a significant reduction in computational memory (3-fold) and processing time (9-fold) [7]. Using an ordinary laptop computer, our motif identifier was able to generate the 20-mer HPt motif in under five minutes, and our motif searcher was able to scan the *A*. *thaliana* and *S*. *pombe* proteomes and generate a boxplot in less than one minute (All sample input files are available in the aforementioned repository). Based on runtime values, the time cost of the motif finder seems to increase linearly with increasing k, so it is also possible to generate much longer motifs in a reasonable amount of time. Probabilistic motif searching of a new proteome or an assemblage of proteomes offers a computationally compact and readily customized means of identifying candidate proteins worthy of subsequent genetic and biochemical investigation. We have demonstrated here that this method performs comparably to those employed by major protein sequence databases, while doing so within a matter of minutes on a user’s local computer.

## Supporting Information

S1 Fig Histone Family Motif Logo. A 15-mer motif profile was generated for the Pfam histone family (PF00125) representative set RP15, 1432 sequences, accessed Nov. 2014.
(TIF)

S2 Fig 3D Representation of the Histone Motif. The Profile most probable 15-mer is highlighted as space-filled spheres on the human nucleosome (PDB ID 3AN2). Highlighted residues are 90–104 on Histone H3-like centromeric protein A (Chains A and E, red), 69–83 on Histone H2B type 1-J (Chains D and H, purple), 54–68 on Histone H2A type 1-B/E (Chains C and G, blue) and 34–48 on Histone H4 (Chains B and F, yellow). DNA is shown in green. Without constraining the motif finder to any specific type of histone, we were able to identify a 15 amino acid region, conserved among all types, that is structurally relevant to both histone-histone interaction and to histone-DNA binding. Nucleosome viewed from the side (A) or the top (B) using the VMD software [32].
(TIF)

S3 Fig Boxplot of ϕ Scores of Histones of Various Proteomes. ϕ Scores of *Mus musculus*, *Homo sapiens*, *Drosophila melanogaster*, *Danio rerio*, *Saccharomyces cerevisiae*, and *Dictyostelium discoideum* proteins are presented. Outliers above the upper 3IQR whisker are classified as positive hits presumptively belonging to the histone family.
(TIF)

S1 Table HPt Hits Comparison in Different Databases. HPt proteins from eleven organisms are presented, as identified by motif searching or as found in any of the databases Pfam, SMART, InterPro, or PROSITE. The unique UniProt ID of each protein is indicated. ‘Motif found as’ indicates the profile-most-probable 20-mer within the protein sequence, followed by its corresponding ϕ (Phi) value; the ϕ value is that computed by the motif finder algorithm (see Methods). “+” indicates that the protein is positively identified as an HPt family member by the database indicated, or as an outlier according to the Motif 3IQR or Motif 5% cutoffs (see Methods).
(XLSX)

S2 Table Histone Hits as Identified by the Motif Finder. For each of six eukaryotic organisms, the protein sequences with the highest ϕ scores are shown; these include all those above the 3 IQR threshold (underlined entries) followed by ten additional sequences. Proteins scoring above the threshold are suggested by the motif method to belong to the histone family. The UniProt annotations demonstrate almost complete overlap between proteins identified by their motif likelihood here and those previously described as histones; similarly, the ten proteins in each proteome just below the motif cutoff are, for the most part, not thought to be members of the histone family. No 5% cutoff is indicated in this table as, for this motif, it is less conservative than the 3IQR threshold shown.
(XLSX)

## References

[1] Stock AM, Robinson VL, Goudreau PN. Two-Component Signal Transduction. Annual Review of Biochemistry. 2000;69(1):183–215. 10.1146/annurev.biochem.69.1.183 10966457

[2] Thomason P, Kay R. Eukaryotic signal transduction via histidine-aspartate phosphorelay. Journal of cell science. 2000;113(18):3141–50. 10954413 10.1242/jcs.113.18.3141

[3] Tekinay T, Ennis HL, Wu MY, Nelson M, Kessin RH, Ratner DI. Genetic Interactions of the E3 Ubiquitin Ligase Component FbxA with Cyclic AMP Metabolism and a Histidine Kinase Signaling Pathway during Dictyostelium discoideum Development. Eukaryotic Cell. 2003 6 1;2(3):618–26. 12796307 10.1128/EC.2.3.618-626.2003 PMC161463

[4] Tsuzuki M, Ishige K, Mizuno T. Phosphotransfer circuitry of the putative multi-signal transducer, ArcB, of Escherichia coli: in vitro studies with mutants. Molecular Microbiology. 1995 12 1;18(5):953–62. 8825099 10.1111/j.1365-2958.1995.18050953.x

[5] Fassler JS, West AH. Histidine Phosphotransfer Proteins in Fungal Two-Component Signal Transduction Pathways. Eukaryotic Cell. 2013 8 1;12(8):1052–60. 10.1128/EC.00083-13 23771905 PMC3754533

[6] Ishige K, Nagasawa S, Tokishita S, Mizuno T. A novel device of bacterial signal transducers. EMBO J. 1994 11 1;13(21):5195–202. 7957084 10.1002/j.1460-2075.1994.tb06850.x PMC395468

[7] Eddy SR. Profile hidden Markov models. Bioinformatics. 1998 1 1;14(9):755–63. 9918945 10.1093/bioinformatics/14.9.755

[8] Geman S, Geman D. Stochastic Relaxation, Gibbs Distributions, and the Bayesian Restoration of Images. IEEE Transactions on Pattern Analysis and Machine Intelligence. 1984 11;PAMI-6(6):721–41. 10.1109/tpami.1984.4767596 22499653

[9] Finn RD, Bateman A, Clements J, Coggill P, Eberhardt RY, Eddy SR, et al Pfam: the protein families database. Nucl Acids Res. 2014 1 1;42(D1):D222–D230. 24288371 10.1093/nar/gkt1223 PMC3965110

[10] Consortium TU. UniProt: a hub for protein information. Nucl Acids Res. 2015 1 28;43(D1):D204–D212. 25348405 10.1093/nar/gku989 PMC4384041

[11] Mourey L, Re SD, Pédelacq J-D, Tolstykh T, Faurie C, Guillet V, et al Crystal Structure of the CheA Histidine Phosphotransfer Domain that Mediates Response Regulator Phosphorylation in Bacterial Chemotaxis. J Biol Chem. 2001 8 17;276(33):31074–82. 11387324 10.1074/jbc.M101943200

[12] Sugawara H, Kawano Y, Hatakeyama T, Yamaya T, Kamiya N, Sakakibara H. Crystal structure of the histidine-containing phosphotransfer protein ZmHP2 from maize. Protein Sci. 2005 1;14(1):202–8. 15576555 10.1110/ps.041076905 PMC2253335

[13] Song HK, Lee JY, Lee MG, Moon J, Min K, Yang JK, et al Insights into eukaryotic multistep phosphorelay signal transduction revealed by the crystal structure of Ypd1p from Saccharomyces cerevisiae1. Journal of Molecular Biology. 1999 11 5;293(4):753–61. 10543964 10.1006/jmbi.1999.3215

[14] Schneider TD, Stephens RM. Sequence logos: a new way to display consensus sequences. Nucleic Acids Res. 1990 10 25;18(20):6097–100. 2172928 10.1093/nar/18.20.6097 PMC332411

[15] Crooks GE, Hon G, Chandonia J-M, Brenner SE. WebLogo: a sequence logo generator. Genome research. 2004;14(6):1188–90. 15173120 10.1101/gr.849004 PMC419797

[16] Suzuki T, Imamura A, Ueguchi C, Mizuno T. Histidine-Containing Phosphotransfer (HPt) Signal Transducers Implicated in His-to-Asp Phosphorelay in Arabidopsis. Plant Cell Physiol 1998 12; 39(12):1258–68. 10050311 10.1093/oxfordjournals.pcp.a029329

[17] Jung KW, Oh S-I, Kim YY, Yoo KS, Shin MHC and JS. Arabidopsis Histidine-containing Phosphotransfer Factor 4 (AHP4) Negatively Regulates Secondary Wall Thickening of the Anther Endothecium during Flowering. Mol Cells 2008 4 30; 25 (2):294–300. 18413999

[18] Miyata S, Urao T, Yamaguchi-Shinozaki K, Shinozaki K. Characterization of genes for two-component phosphorelay mediators with a single HPt domain in Arabidopsis thaliana. FEBS Letters 1998 10 16;437(1–2):11–4. 9804162 10.1016/s0014-5793(98)01188-0

[19] Mähönen AP, Bishopp A, Higuchi M, Nieminen KM, Kinoshita K, Törmäkangas K, et al Cytokinin signaling and its inhibitor AHP6 regulate cell fate during vascular development. Science. 2006 1 6;311(5757):94–8. 16400151 10.1126/science.1118875

[20] Chang W-T, Thomason PA, Gross JD, Newell PC. Evidence that the RdeA protein is a component of a multistep phosphorelay modulating rate of development in Dictyostelium. The EMBO Journal. 1998;17(10):2809–16. 9582274 10.1093/emboj/17.10.2809 PMC1170621

[21] Mizuno T. His-Asp phosphotransfer signal transduction. Journal of biochemistry. 1998;123(4):555–63. 9538242 10.1093/oxfordjournals.jbchem.a021972

[22] Moreira S, Bishopp A, Carvalho H, Campilho A. AHP6 inhibits cytokinin signaling to regulate the orientation of pericycle cell division during lateral root initiation. PLoS ONE. 2013;8(2):e56370 10.1371/journal.pone.0056370 23457561 PMC3572949

[23] Schaller GE, Shiu S-H, Armitage JP. Two-Component Systems and Their Co-Option for Eukaryotic Signal Transduction. Current Biology. 2011 5 10;21(9):R320–R330. 10.1016/j.cub.2011.02.045 21549954

[24] Besant PG, Attwood PV. Mammalian histidine kinases. Biochim Biophys Acta. 2005 12 30;1754(1–2):281–90. 16188507 10.1016/j.bbapap.2005.07.026

[25] Hutchison CE, Li J, Argueso C, Gonzalez M, Lee E, Lewis MW, et al The Arabidopsis histidine phosphotransfer proteins are redundant positive regulators of cytokinin signaling. Plant Cell. 2006 11;18(11):3073–87. 17122069 10.1105/tpc.106.045674 PMC1693944

[26] Sweere U, Eichenberg K, Lohrmann J, Mira-Rodado V, Bäurle I, Kudla J, et al Interaction of the response regulator ARR4 with phytochrome B in modulating red light signaling. Science. 2001 11 2;294(5544):1108–11. 11691995 10.1126/science.1065022

[27] Hall BP, Shakeel SN, Amir M, Ul Haq N, Qu X, Schaller GE. Histidine kinase activity of the ethylene receptor ETR1 facilitates the ethylene response in Arabidopsis. Plant Physiol. 2012 6;159(2):682–95. 10.1104/pp.112.196790 22467798 PMC3375934

[28] Schultz J, Milpetz F, Bork P, Ponting CP. SMART, a simple modular architecture research tool: Identification of signaling domains. PNAS. 1998 5 26;95(11):5857–64. 9600884 10.1073/pnas.95.11.5857 PMC34487

[29] Mitchell A, Chang H-Y, Daugherty L, Fraser M, Hunter S, Lopez R, et al The InterPro protein families database: the classification resource after 15 years. Nucl. Acids Res. 2014 11 26;gku1243. 10.1093/nar/gku1243 PMC4383996 25428371

[30] Sigrist CJA, de Castro E, Cerutti L, Cuche BA, Hulo N, Bridge A, et al New and continuing developments at PROSITE. Nucleic Acids Res. 2013 1;41(Database issue):D344–7. 10.1093/nar/gks1067 23161676 PMC3531220

[31] Hunter S, Jones P, Mitchell A, Apweiler R, Attwood TK, Bateman A, et al InterPro in 2011: new developments in the family and domain prediction database. Nucl Acids Res. 2012 1 1;40(D1):D306–D312. 22096229 10.1093/nar/gkr948 PMC3245097

[32] Humphrey W, Dalke A, Schulten K. VMD: visual molecular dynamics. J Mol Graph. 1996 2;14(1):33–8, 27–8. 8744570 10.1016/0263-7855(96)00018-5

[33] Podust LM, Ioanoviciu A, Ortiz de Montellano PR. 2.3 Å X-ray Structure of the Heme-Bound GAF Domain of Sensory Histidine Kinase DosT of Mycobacterium tuberculosis. Biochemistry. 2008 11 25;47(47):12523–31. 10.1021/bi8012356 18980385 PMC2645934

[34] Thompson W, Rouchka EC, Lawrence CE. Gibbs Recursive Sampler: finding transcription factor binding sites. Nucleic Acids Res. 2003 7 1;31(13):3580–5. 12824370 10.1093/nar/gkg608 PMC169014

[35] Thijs G, Marchal K, Lescot M, Rombauts S, De Moor B, Rouzé P, et al A Gibbs sampling method to detect overrepresented motifs in the upstream regions of coexpressed genes. J Comput Biol. 2002;9(2):447–64. 12015892 10.1089/10665270252935566

[36] Eddy SR. Accelerated Profile HMM Searches. PLoS Comput Biol [Internet]. 2011 10 20 [cited 2015 Nov 17];7(10):e1002195 Available from: 10.1371/journal.pcbi.1002195 PMC3197634 22039361

[37] Basu S, Fey P, Pandit Y, Dodson R, Kibbe WA, Chisholm RL. dictyBase 2013: integrating multiple Dictyostelid species. Nucl Acids Res. 2013 Jan 1;41(D1):D676–D683. 23172289 10.1093/nar/gks1064 PMC3531180

