# Supplementary figures and images for: Use of a Probabilistic Motif Search to Identify Histidine Phosphotransfer Domain-Containing Proteins
Source: PLoS One. 2016 Jan 11;11(1):e0146577. doi: 10.1371/journal.pone.0146577 (PMC4709007; doi:10.1371/journal.pone.0146577)

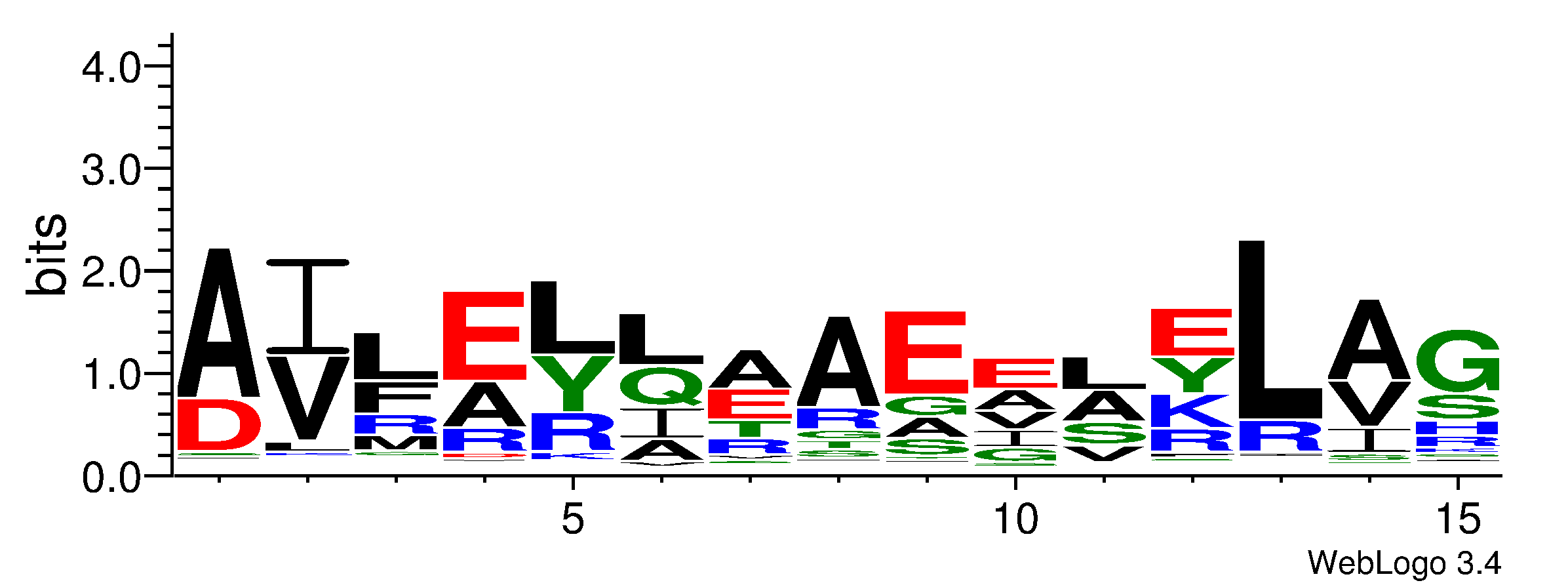

Supplement: S1 Fig — A 15-mer motif profile was generated for the Pfam histone family (PF00125) representative set RP15, 1432 sequences, accessed Nov. 2014. (TIF) [file pone.0146577.s001.tif]

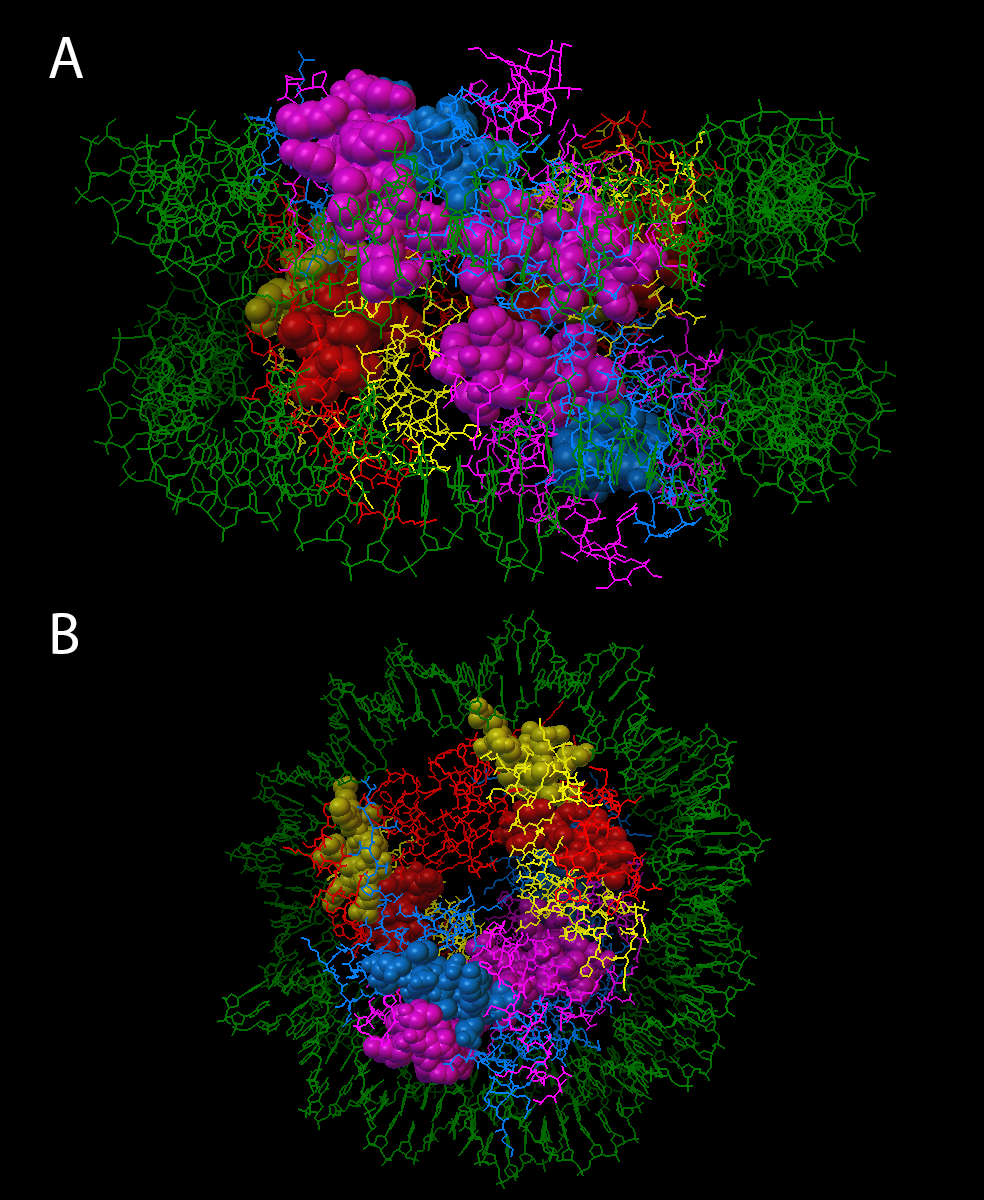

Supplement: S2 Fig — The Profile most probable 15-mer is highlighted as space-filled spheres on the human nucleosome (PDB ID 3AN2). Highlighted residues are 90–104 on Histone H3-like centromeric protein A (Chains A and E, red), 69–83 on Histone H2B type 1-J (Chains D and H, purple), 54–68 on Histone H2A type 1-B/E (Chains C and G, blue) and 34–48 on Histone H4 (Chains B and F, yellow). DNA is shown in green. Without constraining the motif finder to any specific type of histone, we were able to identify a 15 amino acid region, conserved among all types, that is structurally relevant to both histone-histone interaction and to histone-DNA binding. Nucleosome viewed from the side (A) or the top (B) using the VMD software [32]. (TIF) [file pone.0146577.s002.tif]

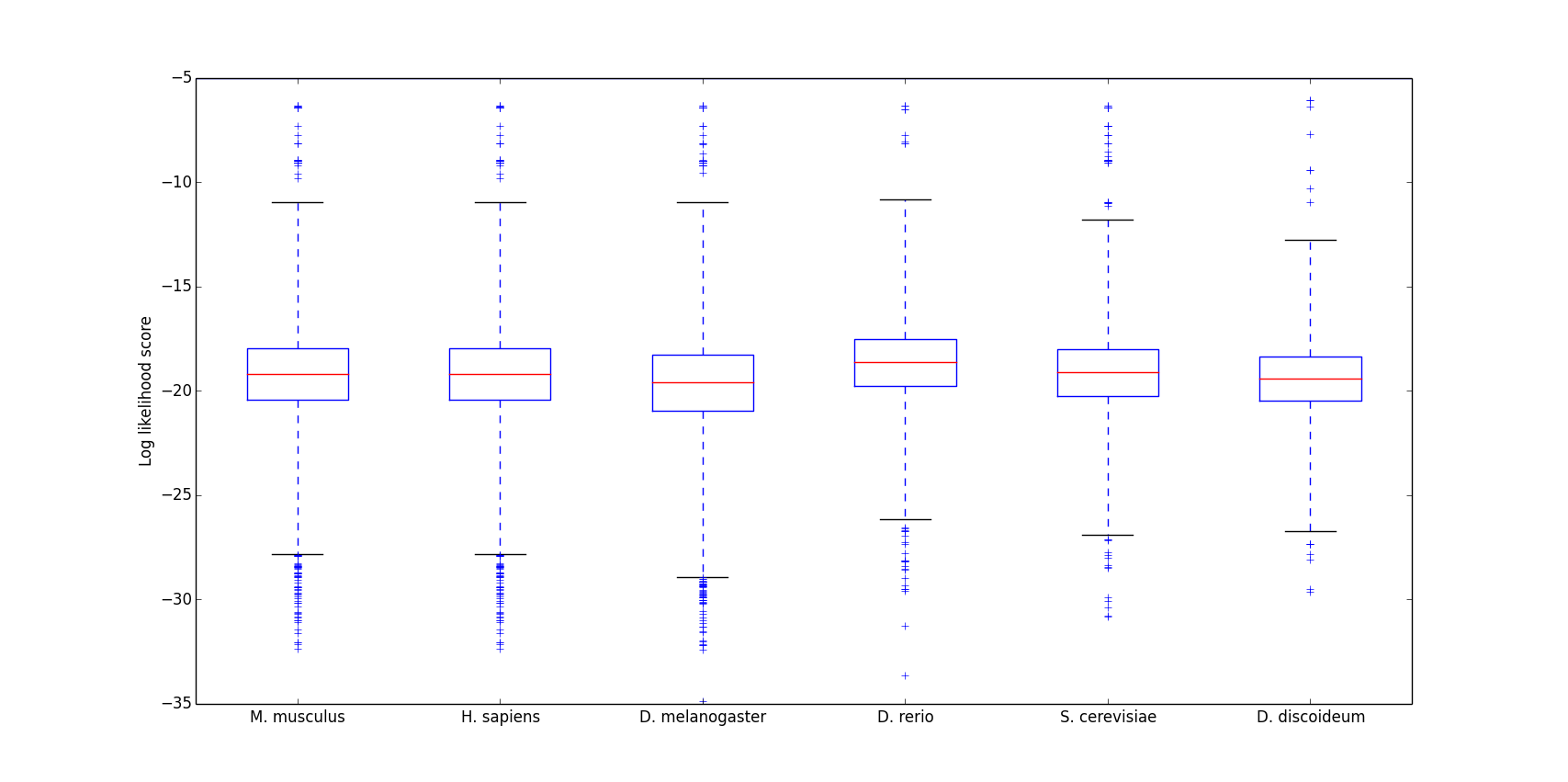

Supplement: S3 Fig — ϕ Scores of Mus musculus, Homo sapiens, Drosophila melanogaster, Danio rerio, Saccharomyces cerevisiae, and Dictyostelium discoideum proteins are presented. Outliers above the upper 3IQR whisker are classified as positive hits presumptively belonging to the histone family. (TIF) [file pone.0146577.s003.tif]
